# A Clinical Practice Example of Smith–Magenis Syndrome in the Neuropediatric Clinic: Etiology, Clinical Presentation, Diagnostics and Therapeutic Approaches—A Case Report

**DOI:** 10.3390/children13020179

**Published:** 2026-01-28

**Authors:** Oleksandr Shevchenko

**Affiliations:** Center for Children and Adolescents Inn-Salzach-Rott, 84503 Altoetting, Germany; o.shevchenko@kinderzentrum.de

**Keywords:** Smith-Magenis syndrome, 17p11.2 deletion, RAI1, neurogenetics, sleep disorder, developmental disorder, EEG, neuropediatrics

## Abstract

**Highlights:**

**What are the main findings?**

- Smith–Magenis syndrome (SMS) presents with a characteristic constellation of neurodevelopmental, behavioral, and sleep–wake disturbances, making early clinical recognition essential for accurate diagnosis.
- A multidisciplinary diagnostic approach—including genetic testing (deletion 17p11.2 or RAI1 mutations), developmental evaluation, and behavioral assessment—is crucial for establishing the diagnosis and guiding individualized therapy.

**What are the implication of the main findings?**

- Early identification of SMS allows for a timely initiation of targeted therapeutic interventions (behavioral management, sleep regulation, parent support), which can significantly improve the quality of life for patients and families.
- The case report underscores the importance of collaboration among neuropediatricians, geneticists, psychologists, and sleep specialists to meet the complex clinical needs of individuals with SMS.
- “Rolando-type” spike-and-sharp wave complexes (benign epilepsy-type potentials of childhood, BEPC) in the EEG are a specific marker of brain immaturity in children and have two main causes: a genetic predisposition with congenital impairment of brain maturation, and organic lesions of the central nervous system. In this case (Smith–Magenis syndrome (SMS)), there is also a congenital disorder of brain maturation, manifested by the presence of benign epilepsy-type potentials of childhood (BEPC, “Rolando-type” spike-and-sharp waves) in the EEG. This study (paper) presents and describes “Rolando-type” spike-and-sharp wave complexes on the EEG in Smith–Magenis syndrome for the first time.

**Abstract:**

Background/Objectives: Smith–Magenis syndrome (SMS) is a rare neurogenetic disorder caused by a microdeletion in chromosome region 17p11.2 or by pathogenic variants in the RAI1 gene. The syndrome is characterized by a distinctive neurobehavioral profile, including cognitive deficits, sleep disturbances, self-injurious behavior, and typical dysmorphic features. A characteristic diagnostic hallmark is paradoxical melatonin secretion, with increased daytime levels instead of the normal nocturnal peak. This article aims to summarize current knowledge on the etiology, diagnostics, EEG findings, therapy, and prognosis of SMS from a neuropediatric perspective. Methods: A narrative review of the literature on Smith–Magenis syndrome was conducted, focusing on genetic background, clinical features, diagnostic approaches, EEG characteristics, therapeutic strategies, and prognosis. In addition, a detailed clinical case of a 16-year-old female patient with SMS is presented. Results: The reviewed data confirm that SMS is associated with characteristic neurobehavioral abnormalities and sleep–wake rhythm disturbances. EEG findings may include epileptiform activity without overt epilepsy. In the presented case, “Rolandic-type” spike–sharp wave complexes were observed on EEG and are interpreted as an expression of congenital disturbances in brain maturation processes. Therapeutic recommendations addressing behavioral symptoms and sleep regulation are discussed. Conclusions: Smith–Magenis syndrome represents a complex neurodevelopmental disorder with distinctive clinical, neurophysiological, and genetic features. Early recognition of characteristic signs, including sleep disturbances and EEG abnormalities, is essential for appropriate management. A multidisciplinary, individualized therapeutic approach may improve quality of life and long-term outcomes.

## 1. Introduction

The discovery of Smith–Magenis syndrome is closely linked to the work of A.C. Smith and E. Magenis. In 1982, they first described two patients with lip and facial clefts and a deletion of the short arm of chromosome 17 [1].

In 1986, a more detailed description of a group of nine unrelated children with a deletion of the 17p11.2 locus was published; these children exhibited a specific clinical phenotype [2]. As early as 1984, a case of a child with an interstitial deletion in the 17p11.2 locus had been reported.

The four-year-old patient presented with intellectual and speech developmental delay, hypotonia, small auricles, hearing impairment, dental enamel dysplasia, and a prominent maxilla [3].

In 1990, five additional cases were described at the Royal Children’s Hospital in Melbourne (Australia); this publication was the first to use the term “Smith–Magenis syndrome” [4].

Greenberg et al. (1991) finally concluded that Smith–Magenis syndrome is a hereditary genetic deletion syndrome [5].

In 2003, Walz et al. developed an animal model of Smith–Magenis syndrome by studying mice with a specific Df(11)17 deletion. The heterozygous deletion of this chromosomal segment led to craniofacial malformations, obesity, epileptic seizures, behavioral abnormalities, and reduced fertility in male animals [6].

This syndrome is one of the rare but clinically characteristic neurogenetic syndromes that are increasingly being diagnosed in neuropediatric centers. The combination of cognitive developmental disorder, pronounced sleep disturbances, self-injurious behavior, and a specific facial appearance makes Smith–Magenis syndrome an important differential diagnostic candidate in complex neurodevelopmental disorders.

A particularly important diagnostic feature is the inverted (“paradoxical”) secretion of melatonin—a daytime increase instead of the typical nocturnal peak. In addition, there is evidence of disturbed light/dark responses and dysfunction of the melanopsin/ipRGC system, which contributes to the so-called “paradoxical light reactions” [7].

The melanopsin/ipRGC system consists of intrinsically photosensitive retinal ganglion cells (ipRGCs) that contain the light-sensitive protein melanopsin and function as a kind of third photoreceptor alongside rods and cones. Melanopsin is a photoreceptive protein present in these cells, enabling them to respond to light independently of other photoreceptors. This melanopsin/ipRGC system is primarily responsible for sensing ambient brightness rather than forming visual images. Its main functions are the synchronization of the circadian pacemaker (the internal clock), control of the pupillary reflex, and regulation of melatonin production [8,9,10].

## 2. Etiology and Genetics: Genotype–Phenotype Correlations

In more than 90% of cases, Smith–Magenis syndrome is caused by a microdeletion of 17p11.2 that includes the Retinoic Acid Induced 1 (RAI1) gene. In approximately 10%, a pathogenic RAI1 point mutation without deletion is present [11]. The RAI1 gene encodes a nuclear protein involved in the regulation of circadian rhythms, neuronal differentiation, and synaptic plasticity. RAI1 is expressed in the suprachiasmatic nucleus and other hypothalamic regions that regulate circadian rhythm. RAI1 is considered a key factor in the phenotypic features, particularly the neurobehavioral and circadian disturbances [12]. Most deletions arise de novo through non-allelic homologous recombination during meiosis. Sleep/melatonin disturbances occur in almost all patients, regardless of the type of genetic alteration, and are considered a consistent phenotypic marker of the syndrome [13].

Patients with 17p11.2 deletions have Smith–Magenis syndrome caused by a heterozygous interstitial deletion of the short arm of chromosome 17 (region p11.2). This deletion encompasses multiple genes, including RAI1, and results in haploinsufficiency of these genes, which is the most common genetic mechanism underlying Smith–Magenis syndrome.

Patients with RAI1 mutations have Smith–Magenis syndrome caused by pathogenic sequence variants in the RAI1 gene (e.g., nonsense, frameshift, splice-site, or deleterious missense variants) without the chromosomal deletion of 17p11.2. In these cases, the disorder is due to RAI1 haploinsufficiency alone, with the remainder of chromosome 17p11.2 being intact [11,12,13].

Some studies show that in patients with deletions, not only is RAI1 affected but also additional genes within the 17p11.2 region, which contribute to phenotypic variability. Girirajan et al. (2006) demonstrated in a cohort of 31 patients that 21 of 30 features were attributable to RAI1 haploinsufficiency, whereas cardiac malformations, speech/motor delay, hypotonia, short stature, and hearing loss were more frequently associated with larger or atypical deletions [14].

A meta-analysis of 105 cases showed significant differences between patients with deletions and those with RAI1 mutations, for example regarding overweight, self-injury, cardiac defects, short or tall stature, etc. Patients with an RAI1 mutation exhibited hyperphagia, obesity, self-hugging, muscle cramps, and dry skin more frequently, and were less often affected by short stature, hearing loss, recurrent ear infections, and cardiac defects than patients with deletions. A subgroup of patients with small deletions, spanning the region from TNFRSF13B to MFAP4, showed lower rates of brachycephaly, dental abnormalities, iris anomalies, head-banging behavior, and hyperactivity. Significant sex-related differences were also observed: female patients more frequently displayed myopia, feeding/appetite problems, cold hands and feet, and communication-related frustration than male patients [15].

A more recent study (Linders et al., 2023) involving 66 individuals found that patients with 17p11.2 deletions had lower average IQ levels (median 56 vs. 73.5) compared with those with RAI1 mutations; at the same time, the RAI1-variant group showed higher levels of internalizing behavioral problems [16].

Further exploratory studies demonstrated differences, for example, in body weight and lipid profiles between deletion and mutation patients [17].

These findings show that the clinical presentation of Smith–Magenis syndrome is determined not only by the presence of a deletion or mutation but also by the size and location of the deletion, the involvement of additional genes, as well as environmental and epigenetic factors. Environmental and epigenetic factors have been shown to influence gene expression and phenotypic outcomes through mechanisms such as DNA methylation and histone modification. Early-life exposures, including prenatal stress, maternal nutrition, and pollutants, can leave lasting epigenetic marks that affect neurodevelopment and disease susceptibility, as described in recent human and animal studies [18,19,20].

This means that diagnostics must be performed as precisely as possible (e.g., using molecular genetic methods) and that management should be individually tailored to the respective genotype/phenotype. Genetic testing should be performed early. This is necessary to confirm the diagnosis, provide accurate genetic counseling, and inform the family about the risk of disease recurrence. Even though the majority of SMS cases arise de novo, rare instances of inherited mutations, parental mosaicism, or structural chromosome rearrangements affecting 17p11.2 have been documented. This means that unaffected or mildly affected parents may carry genetic alterations that increase the recurrence risk in future pregnancies. In such cases, family testing can alter risk estimates significantly and guide reproductive planning.

Importantly, once the specific genetic alteration in the proband is identified, prenatal and preimplantation genetic testing become available options for future pregnancies, allowing families to make informed reproductive decisions. Early diagnosis also enables a timely intervention with multidisciplinary care (e.g., developmental support, behavioral therapy, sleep management), which can improve long-term outcomes [21,22].

## 3. Prevalence and Epidemiology

Smith–Magenis syndrome is classified as a rare disease, with an estimated prevalence of approximately 1:15,000 to 1:25,000 live births [23,24]. With the increasing use of genome-wide array and sequencing techniques, higher detection rates of mild or atypical forms (especially RAI1 mutations) have been reported [25]. Due to underdiagnosis, the actual number of affected individuals is assumed to be higher than the documented cases.

The sex ratio is about 1:1, and cases have been reported worldwide. The syndrome occurs in all ethnic groups and countries. There is no evidence that parental age influences the frequency of the deletion.

## 4. Paradoxical Melatonin Secretion and Light Responses in Smith–Magenis Syndrome

Melatonin (“sleep hormone”) regulates the sleep–wake cycle. It is produced in the pineal gland and helps reduce sleep onset time. Typically, melatonin levels rise in the evening/night, peak during the night, and decline again in the morning. Light suppresses melatonin production via retinohypothalamic signaling to the SCN (suprachiasmatic nucleus).

In patients with Smith–Magenis syndrome, an inversion of the melatonin rhythm has been observed. The rise in melatonin begins in the morning (~6 a.m.), reaches its peak at midday (~12 p.m.), and decreases in the evening (~8 p.m.). De Leersnyder et al. (2003) reported precisely these characteristics in 20 children with Smith–Magenis syndrome [26].

A study assessing urinary excretion of 6-sulfatoxymelatonin in 19 patients showed circadian rhythm disturbances in all but 1 patient [12].

The exact mechanisms underlying this “paradoxical” melatonin secretion remain unclear. However, several hypotheses and explanations have been proposed:

- Haploinsufficiency of RAI1 and/or other genes in 17p11.2 that influence circadian regulatory mechanisms. Haploinsufficiency disorders are genetic conditions caused by reduced gene expression and lead to developmental, metabolic, and tumor-related abnormalities. The dosage-sensitive Retinoic Acid Induced 1 (RAI1) gene, located in the 17p11.2 region, is central to the core features of Smith–Magenis syndrome [27].
- Impairment of melatonin production, secretion, or metabolism [12].
- Absent or reduced response to daylight or disturbed light/dark synchronization [28].

This altered melatonin rhythm explains the typical sleep–wake problems in patients with Smith–Magenis syndrome: early sleep onset, early morning awakening, daytime sleepiness, and nighttime awakening essentially reflect the core sleep–wake disturbances.

An important component of circadian regulation is light perception via intrinsically photosensitive retinal ganglion cells (ipRGCs) containing the photopigment melanopsin. These cells respond primarily to blue light (~470 nm) and influence melatonin suppression as well as the synchronization of the SCN [29].

A study in patients with Smith–Magenis syndrome demonstrated an altered pupillary light reflex (PLR) to blue light: the sustained component was reduced, meaning that the pupil returned to baseline more quickly after a 470 nm flash compared to controls [30]. These findings may be described as “paradoxical,” given that high light exposure during the day should suppress melatonin. In patients with Smith–Magenis syndrome, however, this response is impaired—potentially contributing to the paradoxical daytime melatonin secretion. In addition, a misalignment of the internal clock may be present, preventing correct responses to light/dark cues.

The evidence of disrupted ipRGC/melanopsin function suggests that light-therapy strategies and environmental light design may play an important role in SMS management—for example: early morning daylight exposure, reduction in artificial light in the evening, and targeted control of blue-light exposure. In combination with melatonin therapy and pharmacological interventions, these measures may improve the sleep–wake rhythm [31].

## 5. Clinical and Neurological Manifestations

Smith–Magenis syndrome is a multisystem neurobehavioral disorder with a characteristic developmental and behavioral profile (Table 1).

## 6. Diagnostics

### 6.1. Genetic Diagnostics

- Array-CGH or MLPA to detect a 17p11.2 deletion;
- Sequencing of the *RAI1* gene if no deletion is detectable;
- Confirmation by FISH or SNP array in unclear cases.

### 6.2. EEG Findings

EEG changes are nonspecific. Common findings include the following:

- Intermittent frontocentral slowing;
- Absence of epileptiform activity despite behavioral abnormalities;
- In approximately 10–20% of cases, generalized or focal epileptiform potentials may occur [32].

### 6.3. Sleep Diagnostics

Polysomnography and melatonin profiling confirm the inverted circadian rhythm, which is diagnostically and therapeutically relevant [33].

**Case Report: Girl, 16 Years Old**

A sixteen-year-old patient (clinical, neuropediatric, orthopedic, psychological, genetic, EEG, echocardiography, ECG, and hearing assessments) in whom Smith–Magenis syndrome has now been genetically confirmed. EEG recordings were performed using silver-chloride electrodes according to the 10–20 electrode system in accordance with the guidelines of the DGKN (German Society for Clinical Neurophysiology and Functional Imaging—Recommendations for EEG Recording Protocols). Bipolar longitudinal montage was used (for better spatial resolution and clearer visualization of focal EEG findings). Gain: 7–10 µV. Time constant (high-pass filter): 0.3 s (0.5 Hz). Low-pass filter: 70 Hz (EEG system: Micromed).

Pregnancy and birth were unremarkable. The patient was born at 41 weeks of gestation weighing 3800 g (70th percentile), length 52 cm (46th percentile), weight/length ratio 73.1 kg/cm (76th percentile). Postnatally, there was a nuchal cord without signs of peripartal asphyxia; the patient was discharged quickly. First independent steps were taken at 11 months. Fine motor difficulties were noted.

**Current Examination Findings:**

Weight: 49.6 kg (16th percentile), height: 163.5 cm (35th percentile), head circumference: 55 cm (36th percentile), BMI: 18.6 kg/m^2^ (21st percentile).

The 16-year-old girl is in good general and nutritional condition. Slender habitus.

**Facial features:** Prognathism, oligodontia, and broad nose. Malocclusion; status post-surgical tooth extraction. Hands with short and broad fingers, particularly the thumbs. Syndactyly of toes II and III on both sides.

**Spine/Orthopedic:** Pronounced idiopathic adolescent scoliosis; status post thoracolumbar spondylodesis (2022) and long-segment spondylodesis TH2-L5 (2024). Scar from spondylodesis is unremarkable along the entire back. Shoulder girdle is tense, right scapula prominent, waist remains asymmetrical.

**Skin:** Rosy complexion, juvenile acne.

**Respiratory:** Symmetrical breath sounds, no obstruction.

**Cardiovascular:** Heart rhythm regular, no systolic murmur. Pulses palpable and normal.

**Abdomen:** Soft, non-tender, no masses, no hepatosplenomegaly. No ascites, no edema.

**Neurological:** Unremarkable.

**Psychological and Developmental Neurology Findings:**

Mild intellectual disability without behavioral abnormalities (total IQ 60). Expressive and receptive speech disorder, dysarthria, limited sentence formation, focal developmental disorder of motor functions. Attends a special education center for intellectual development.

**Eyes and Ears:**

Left-sided amblyopia (30% visual acuity), normal hearing.

**Hearing Assessment:** In free-field testing with frequency-specific sounds, appropriate orientation to the corresponding speaker was observed at a sound level of 35–40 dB in the 1000–12,500 Hz frequency range.

**Impedance:** Normal middle ear pressure bilaterally.

**Transient Evoked Otoacoustic Emissions (T-OAEs):** Clearly positive bilaterally. Frequency response spectrum: 500–5000 Hz.

**Cardiovascular:**

Arterial hypertension grade I (treated with valsartan since 2024, currently 40-0-20 mg), history of pulmonary hypertension.

**General:**

Toilet training completed. Nutrition and digestion unremarkable.

Increased susceptibility to infections, with frequent recurrent pulmonary infections due to restricted respiratory excursion caused by scoliosis.

**Sleep Behavior:** Pronounced early riser (4–5 a.m.), no seizures.

**Sleep Quality Questionnaire (Pittsburgh Sleep Quality Index, PSQI, German version, German Society for Sleep Research and Sleep Medicine) [34,35,36]**:

Questionnaire assessing sleep quality over the past four weeks (including frequency of sleep-disturbing events, subjective sleep quality, usual sleep times, sleep latency and duration, use of sleep medication, and daytime sleepiness). A total of 18 items are used for quantitative evaluation and are assigned to 7 components, each with a score range of 0–3. The total score is obtained by summing the component scores and can range from 0 to 21, with higher scores indicating poorer sleep quality. A score above 5 points indicates poor sleep quality (Table 2).

**Total:** 10 points.

**Conclusion:** Poor sleep quality. For definitive assessment of sleep disturbances, polysomnography with video recording in a pediatric sleep laboratory is necessary.

**Pediatric Sleep Questionnaire—Sleep-Disordered Breathing Subscale (German version 1.0, German Society for Sleep Research and Sleep Medicine)** [35,37].

**Purpose:** To identify potential patients with OSA (Obstructive Sleep Apnea).

**Formula for identifying potential OSA patients:**

Number of “Yes” answers ÷ (Number of “Yes” + “No” answers) = 5 ÷ (5 + 15) = 5 ÷ 20 = 0.25 → No suspicion of OSA.

**Interpretation:**

- Value > 0.33 → Suspicion of OSA (Obstructive Sleep Apnea);
- Value < 0.33 → No suspicion of OSA (Obstructive Sleep Apnea).

**Sleep Questionnaire “Child Sleep Comic” (abnormal findings) (German version, German Society for Sleep Research and Sleep Medicine) [35,38]**:

Maximum score: 20 points. The higher the score, the more severe the sleep problem.

**Abnormal responses:**

- “What do you do when you cannot fall asleep?” → “I bring my pet into bed.”
- “What do you do when you wake up at night?” → “I wake up because I am hungry or thirsty.”
- “Do you take a nap during the day?” → “Yes.”

**Polysomnography** demonstrates reduced total sleep time, prolonged sleep and REM latency, decreased REM sleep percentage, marked sleep fragmentation with an elevated arousal index, and frequent nocturnal movements. Respiratory parameters are within normal limits. The findings are consistent with the characteristic sleep architecture disturbances observed in Smith–Magenis syndrome.

**Below are the polysomnography data of this patient:**

**Sleep Continuity:**

**Total Sleep Time (TST):** 330 min (5.5 h) (reduced for age)

**Sleep Onset Latency:** 65 min (prolonged)

**Wake After Sleep Onset (WASO):** 95 min (markedly increased; fragmented sleep)

**Sleep Efficiency:** 69% (reduced)

**REM Sleep:**

**REM Sleep Latency:** 150 min (prolonged)

**REM Sleep (% of TST):** 16% (reduced; age norm ≈ 20–25%)

**Non-REM Sleep Distribution:**

**N1:** 14% (increased)

**N2:** 56% (increased)

**N3 (Slow-Wave Sleep):** 14% (reduced)

**Sleep Architecture:**

**Sleep Cycles Completed:** 3 (incomplete, reduced cycling)

**REM Periods:** 2 (short, fragmented; abnormal REM organization)

**Frequent Stage Shifts:** Present

**Arousals:**

**Arousal Index:** 22/h (elevated)

**Type:** Predominantly spontaneous and movement-related

**Respiratory Parameters:**

Apnea–Hypopnea Index (AHI): normal

Oxygen Saturation (SpO_2_): Mean: 95–98%

**Melatonin assessment at 07:00 a.m.:**

Melatonin (venous blood, radioimmunoassay [RIA]): 57 pg/mL.

Reference range: daytime < 30 pg/mL, nighttime < 150 pg/mL.

Urinary melatonin sulfate (enzyme immunoassay): 18 µg/L.

These findings support the presence of a circadian rhythm disturbance characteristic of Smith–Magenis syndrome.

**Echocardiography (Echo) and Electrocardiography (ECG):** Good biventricular pump function, normal valve function, currently no pulmonary hypertension. Resting ECG shows no arrhythmias or signs of hypertrophy, no extrasystoles. Long-term blood pressure monitoring shows stable values.

**Figure 1:**
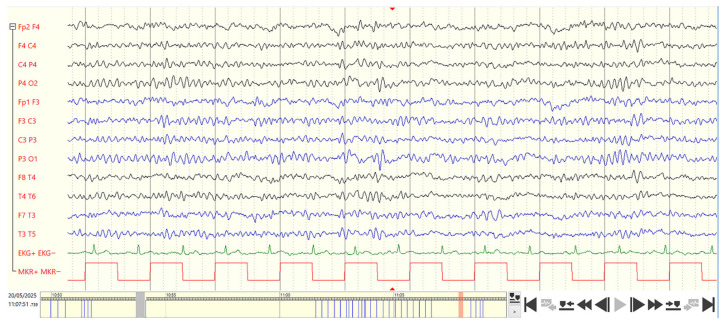
Electroencephalogram (fragment) of a patient with Smith–Magenis syndrome.

**Electroencephalography (EEG):** Bipolar longitudinal montage. Abnormal wake EEG. Five-phasic, monomorphic, and “Rolando-type” spike-and-sharp waves are observed in the posterior-temporal and central regions on both the right and left sides, spreading anteriorly without clinical manifestations on video (Figure 1).

**Note:**

“Rolando-type” spike-and-sharp wave complexes (benign epilepsy-type potentials of childhood, BEPC) in the EEG are a specific marker of brain immaturity in children and have two main causes: a genetic predisposition with congenital impairment of brain maturation, and organic lesions of the central nervous system (concept by Prof. H. Doose: “Hereditary Cerebral Maturation Disorder”). In this case, there is also a congenital disorder of brain maturation, manifested by the presence of benign epilepsy-type potentials of childhood (BEPC, “Rolando-type” spike-and-sharp waves) in the EEG.

“Rolando-type” spike-and-sharp wave complexes are characteristic electroencephalographic abnormalities that appear as a five-point electrical dipole with high amplitude. The total duration of the complex is approximately 70–120 ms.

A defining feature of “Rolando-type” spike-and-sharp wave complexes within non-structural epilepsy syndromes is their strictly age-dependent occurrence, with the typical EEG patterns appearing exclusively during childhood, usually between 3 and 15 years of age, and showing spontaneous remission before or during adolescence. This highlights the maturation-dependent nature of these EEG abnormalities.

This paper presents and describes “Rolando-type” spike-and-sharp wave complexes on the EEG in Smith–Magenis syndrome for the first time.

**Human Genetic Examination:** Whole exome sequencing, microarray analysis, karyotyping: Smith–Magenis syndrome (17p12–p11.2, heterozygous, 3.2 Mb), de novo. The finding is consistent with the diagnosis of Smith–Magenis syndrome. The syndrome follows an autosomal-dominant inheritance pattern; the 17p11.2 deletion has a 50% chance of being passed on to offspring.

Parents and two brothers (18 years and 3 years old) are healthy. No evidence of other familial diseases relevant for genetic counseling.

## 7. Conclusions

Smith–Magenis syndrome is an important differential diagnosis in pediatric neurology for developmental disorders and complex behavioral problems. Genetic testing should be performed early. At present, there is no specific therapy for Smith–Magenis syndrome. Management requires a multidisciplinary approach that integrates sleep rhythm, behavior, communication, and family support. Regular follow-up in neuropediatrics, cardiology, orthopedics, psychology, ophthalmology, ENT, and dentistry is necessary in this case. Therapy with valsartan should be continued unchanged.

“Rolando-type” spike-and-sharp wave complexes (benign epilepsy-type potentials of childhood, BEPC) in the EEG in this case reflect congenital disturbances in brain maturation processes. This paper is the first to report and characterize “Rolando-type” spike-and-sharp wave complexes on EEG in Smith–Magenis syndrome.

Polysomnography showed a markedly fragmented sleep architecture with prolonged sleep latency, multiple arousals, reduced REM sleep proportion, and increased motor activity.

During episodes of increased behavioral abnormalities, causes of physical pain related to undiagnosed conditions (e.g., dental infection, ear infections) should always be considered, as affected individuals may not always communicate these adequately.

For a definitive assessment of sleep disturbances, polysomnography with video recording in a pediatric sleep laboratory is necessary. Sleep hygiene guidelines have been provided (German Society for Sleep Research and Sleep Medicine, DGSM). Melatonin (Mellozzan^®^, extended-release tablets) 2 mg was prescribed in the evening, approximately 30 min before bedtime, with subsequent evaluation and dose monitoring.

Continuation of physiotherapy and speech therapy, along with supportive social counseling for the parents, is recommended.

## Figures and Tables

**Table 1:** Typical findings of Smith–Magenis syndrome.

Parameter	Typical Findings
Cognition/Development	Moderate to severe intellectual disability; delayed speech development (receptive > expressive); hypotonia in infancy
Behavior	Sleep disturbances, self-injurious behavior (e.g., nail biting, self-hitting), hyperactivity, impulsive mood swings, social disinhibition
Sleep	Reversed melatonin rhythm (increased daytime secretion, reduced nighttime secretion). Nighttime sleep is light, characterized by frequent awakenings and early morning waking. During the day, there is pronounced sleepiness and short episodes of drowsiness. The inability to maintain an active wake state in the early evening is associated with irritability and temper outbursts. Thus, the disturbance of circadian rhythms does not correlate with the size of the deletion but appears to be directly related to the dysregulation of RAI1-dependent genes involved in the transcription of circadian clock genes (e.g., CLOCK, PER2, BMAL1) [7,24,25].
Facial dysmorphisms	Brachycephaly, broad forehead, deep-set eyes, short nose, wide upper lip, distinctive midface, upward-turned mouth corners, prominent chin
Neurological features	Motor development is delayed, and hypotonia in infancy as well as gross motor coordination disorders are common. Fine motor deficits are also present. Characteristic features include reduced pain sensitivity and diminished temperature perception. Epileptic seizures occur rarely.
Other features	Recurrent and prolonged rhinitis and sinusitis, as well as otitis, are characteristic. Hearing impairment (conductive and sensorineural hearing loss), myopia, scoliosis, and heart and kidney anomalies (including duplication of the renal pelvis and calyces) are observed. Congenital heart defects occur in approximately 37% of patients. In most cases, these involve atrial and ventricular septal defects, mitral valve prolapse, stenosis or regurgitation of the tricuspid and mitral valves, supravalvular pulmonary stenosis, and subvalvular aortic stenosis [11,12].

**Table 2:** Evaluation: total PSQI score.

Component	Score	Interpretation
1. Subjective sleep quality	2	poor
2. Sleep latency	1	-
3. Sleep duration	1	-
4. Sleep efficiency	2	poor
5. Sleep disturbances	1	-
6. Use of sleep medication	0	none
7. Daytime dysfunction	3	poor

## Data Availability

No new data were created or analyzed in this study. Data sharing is not applicable to this article.

## References

[1] Smith A.C., Magenis R.E. (1982). Two patients with facial clefts and deletion of the short arm of chromosome 17. Am. J. Med. Genet..

[2] Smith A.C., McGavran L., Robinson J., Waldstein G., Macfarlane J., Zonona J., Reiss J., Lahr M., Allen L., Magenis E. (1986). Interstitial deletion of (17)(p11.2p11.2) in nine patients. Am. J. Med. Genet..

[3] Patil S.R., Bartley J.A. (1984). Interstitial deletion of the short arm of chromosome 17. Hum. Genet..

[4] Colley A.F., Leversha M.A., Voullaire L.E., Rogers J.G. (1990). Five cases demonstrating the distinctive behavioural features of chromosome deletion 17(p11.2p11.2) (Smith-Magenis syndrome). J. Paediatr. Child Health.

[5] Greenberg F., Guzzetta V., Montes de Oca-Luna R., Magenis R.E., Smith A., Richter S., Kondo I., Dobyns W., Patel P., Lupski J.R. (1991). Molecular analysis of the Smith-Magenis syndrome: A possible contiguous-gene syndrome associated with del(17)(p11.2). Am. J. Hum. Genet..

[6] Walz K., Caratini-Rivera S., Bi W., Fonseca P., Mansouri D.L., Lynch J., Vogel H., Noebels J.L., Bradley A., Lupski J.R. (2003). Modeling del(17)(p11.2p11.2) and dup(17)(p11.2p11.2) Contiguous Gene Syndromes by Chromosome Engineering in Mice: Phenotypic Consequences of Gene Dosage Imbalance. Mol. Cell. Biol..

[7] De Leersnyder H., De Blois M.C., Claustrat B., Romana S., Albrecht U., von Kleist-Retzow J.C., Delobel B., Viot G., Lyonnet S., MD Vekemans M. (2001). Inversion of the circadian rhythm of melatonin in the Smith-Magenis syndrome. J. Pediatr..

[8] Mure L.S., Hatori M., Zhu Q., Demas J., Kim I.M., Nayak S.K., Panda S. (2016). Melanopsin-Encoded Response Properties of Intrinsically Photosensitive Retinal Ganglion Cells. Neuron.

[9] Do M.T.H. (2019). Melanopsin and the Intrinsically Photosensitive Retinal Ganglion Cells: Biophysics to Behavior. Neuron.

[10] Lucio-Enríquez K.R., Rubio-Valles M., Ramos-Jiménez A., Pérez-León J.A. (2025). Human melanopsin (OPN4) gene polymorphisms: A systematic review. Front. Neurosci..

[11] Greenberg F., Lewis R.A., Potocki L., Glaze D., Parke J., Killian J., Murphy M.A., Williamson D., Brown F., Dutton R. (1996). Multidisciplinary clinical study of Smith-Magenis syndrome (deletion 17p11.2). Am. J. Med. Genet..

[12] Potocki L., Shaw C.J., Stankiewicz P., Lupski J.R. (2000). Variability in clinical phenotype despite common chromosomal deletion in Smith-Magenis syndrome. Genet. Med..

[13] Rinaldi B., Villa R., Sironi A., Garavelli L., Finelli P., Bedeschi M.F. (2022). Smith-Magenis Syndrome-Clinical Review, Biological Background and Related Disorders. Genes.

[14] Girirajan S., Vlangos C.N., Szomju B.B., Edelman E., Trevors C.D., Dupuis L., Nezarati M., Bunyan D.J., Elsea S.H. (2006). Genotype-phenotype correlation in Smith-Magenis syndrome: Evidence that multiple genes in 17p11.2 contribute to the clinical spectrum. Genet. Med..

[15] Edelman E.A., Girirajan S., Finucane B., Patel P., Lupski J., Smith A., Elsea S. (2007). Gender, genotype, and phenotype differences in Smith-Magenis syndrome: A meta-analysis of 105 cases. Clin. Genet..

[16] Linders C.C., van Eeghen A.M., Zinkstok J.R., Boogaard M.-J.v.D., Boot E. (2023). Intellectual and Behavioral Phenotypes of Smith-Magenis Syndrome: Comparisons between Individuals with a 17p11.2 Deletion and Pathogenic *RAI1* Variant. Genes.

[17] Houben M.L. (2020). An exploratory study on Smith Magenis syndrome: Differences in weight and lipid profiles between patients with a 17p11.2 deletion and patients with a RAI1 gene mutation. Tijdschr. Artsen Verstand. Gehandicap..

[18] Jirtle R., Skinner M. (2007). Environmental epigenomics and disease susceptibility. Nat. Rev. Genet..

[19] Jubair H. (2025). Epigenetics and Environmental Exposures in Early Neurodevelopment: Implications for Pediatric Neurological Disorders. Sage Open Pediatr..

[20] Kundakovic M., Jaric I. (2017). The Epigenetic Link between Prenatal Adverse Environments and Neurodevelopmental Disorders. Genes.

[21] Smith A.C.M., Berens J., Boyd K.E., Brennan C., Gropman A., Haas-Givler B., Vlangos C., Foster R., Franciskovich R., Girirajan S., Adam M.P., Bick S., Mirzaa G.M., Pagon R.A., Wallace S.E., Amemiya A. (2026). Smith-Magenis Syndrome; 2001 Oct 22 [Updated 2025 May 29]. GeneReviews^®^ [Internet].

[22] Vieira G.H., Rodriguez J.D., Carmona-Mora P., Cao L., Gamba B.F., Carvalho D.R., Duarte A.d.R., Santos S.R., de Souza D.H., DuPont B.R. (2012). Detection of classical 17p11.2 deletions, an atypical deletion and RAI1 alterations in patients with features suggestive of Smith-Magenis syndrome. Eur. J. Hum. Genet..

[23] Elsea S.H., Girirajan S. (2008). Smith-Magenis syndrome. Eur. J. Hum. Genet..

[24] Gropman A.L., Elsea S., Duncan W.C., Smith A.C. (2020). New developments in Smith-Magenis syndrome (del 17p11.2). Curr. Opin. Neurol..

[25] Laje G., Morse R., Richter W., Ball J., Pao M., Smith A.C. (2010). Autism spectrum features in Smith-Magenis syndrome. Am. J. Med. Genet. C Semin. Med. Genet..

[26] De Leersnyder H., Bresson J.L., de Blois M.C., Souberbielle J.-C., Mogenet A., Delhotal-Landes B., Salefranque F., Munnich A. (2003). Beta 1-adrenergic antagonists and melatonin reset the clock and restore sleep in a circadian disorder, Smith-Magenis syndrome. J. Med. Genet..

[27] Covarelli J., Vinciarelli E., Mirarchi A., Prontera P., Arcuri C. (2025). Retinoic Acid Induced 1 and Smith-Magenis Syndrome: From Genetics to Biology and Possible Therapeutic Strategies. Int. J. Mol. Sci..

[28] Boudreau E.A., Johnson K.P., Jackman A.R., Blancato J., Huizing M., Bendavid C., Jones M., Chandrasekharappa S.C., Lewy A.J., Smith A.C. (2009). Review of disrupted sleep patterns in Smith-Magenis syndrome and normal melatonin secretion in a patient with an atypical interstitial 17p11.2 deletion. Am. J. Med. Genet. A.

[29] Poisson A., Nicolas A., Bousquet I., Raverot V., Gronfier C., Demily C. (2019). Smith-Magenis Syndrome: Molecular Basis of a Genetic-Driven Melatonin Circadian Secretion Disorder. Int. J. Mol. Sci..

[30] Barboni M.T.S., Bueno C., Nagy B.V., Maia P.L., Vidal K.S.M., Alves R.C., Reiter R.J., Amaral F.G.D., Cipolla-Neto J., Ventura D.F. (2018). Melanopsin System Dysfunction in Smith-Magenis Syndrome Patients. Invest. Ophthalmol. Vis. Sci..

[31] Gropman A.L., Duncan W.C., Smith A.C. (2006). Neurologic and Developmental Features of the Smith-Magenis Syndrome (del 17p11.2). Pediatr. Neurol..

[32] Finucane B.M. (2016). Neurologic findings in Smith–Magenis syndrome. Am. J. Med. Genet A.

[33] Gropman A.L., Duncan W.C., Smith A.C. (2022). Neurologic and sleep disturbances in Smith-Magenis syndrome: Integrating circadian rhythm and neurobehavioral phenotypes. Curr. Opin. Psychiatry.

[34] Chervin R.D., Hedger K., Dillon J.E., Pituch K.J. (2000). Pediatric sleep questionnaire (PSQ): Validity and reliability of scales for sleep-disordered breathing, snoring, sleepiness, and behavioral problems. Sleep Med..

[35] Wiater A., Lehmkuhl G. (2011). Handbuch des Kinderschlafs: Grundlagen, Diagnostik und Therapie Organischer und Nicht Organischer Schlafstörungen.

[36] Sagheri D., Wiater A. (2009). Kinderärztlicher Schlaffragebogen (PSQ-DE).

[37] Wessolleck E., Dockter S., Eyth C.P., Lang S., Stuck B.A. (2016). Der Subtest PSQ-SRBD in einer pädaudiologischen Ambulanz. Somnologie.

[38] Schwerdtle B., Kanis J., Kübler A., Schlarb A.A. (2016). The Children’s Sleep Comic: Psychometrics of a Self-rating Instrument for Childhood Insomnia. Child Psychiatry Hum. Dev..

